# Clinical Supervision in Nursing and Evidence‐Based Practices in Radial Haemostasis: A Pilot Study

**DOI:** 10.1155/jonm/6108309

**Published:** 2026-10-15

**Authors:** David Rodrigues, Cristina Barroso Pinto, Paulo Marques

**Affiliations:** ^1^ Department of Cardiology Santo Antonio University Hospital Center ULS Santo Antonio Porto Portugal; ^2^ RISE-Health Nursing School University of Porto Porto Portugal up.pt

**Keywords:** clinical supervision, coronary angiography, haemostasis, nursing care, protocol adherence, radial artery

## Abstract

**Aim:**

To explore radial artery haemostasis practices in relation to recommended protocols, alongside the strategies and characteristics of clinical supervision among nurses providing this care.

**Background:**

The radial artery is the preferred access route for coronary angiography, but successful outcomes depend on appropriate postprocedure haemostasis management, primarily a nursing responsibility. Clinical supervision supports professional development and care quality, yet little is known about its relationship to haemostasis protocols in interventional cardiology settings.

**Methods:**

A descriptive, cross‐sectional study used an online questionnaire distributed among nurses from five Portuguese hospital centres performing coronary angiography via the radial artery, covering demographic data, haemostasis practices and clinical supervision strategies, analysed descriptively.

**Results:**

Among 96 nurses (65.6% female, mean age: 43.2 years, mean experience: 20.7 years), the TR Band device was the predominant haemostasis method, but full adherence to its manufacturer protocol was low (only 6.3% full compliance; 44.2% partial compliance). Partial compliance was similar regardless of cardiology experience, above or below 5 years (42.7% vs 50%). Substantial variability existed in postprocedure care, such as driving restrictions (24 h: 44.8%; 48 h: 47.9%) and puncture site management, with limited written materials provided (24.0%). Regarding clinical supervision, 88.5% lacked formal training. Supervisors favoured explanation and demonstration (87.3%) over feedback (45.1%) and discussion (43.1%). Time constraints and frequent job turnover were the main supervision barriers.

**Conclusion:**

Considerable variation in adherence to the TR Band manufacturer’s protocol was low and variable, underscoring the need for standardised nursing practice. Clinical supervision was predominantly didactic, with limited formative approaches and little formal supervisory training.

**Implications for Nursing Management:**

Nurse managers should consider reinforcing standardised haemostasis protocols through training and monitoring, and separately strengthening supervisor preparation and formative supervision approaches. Addressing time constraints and staff turnover may support both areas, though this cross‐sectional study cannot establish that improved supervision enhances haemostasis practice.

## 1. Introduction

The radial artery has become the preferred access route for coronary angiography and percutaneous coronary interventions, with adoption rates reaching 85.7% in Portugal by 2022 [1]. This preference stems from reduced bleeding complications and improved patient comfort compared to femoral access [2]. However, successful radial access outcomes depend heavily on appropriate postprocedure haemostasis management, which is primarily within the nursing domain [3].

Patent haemostasis, first described by Pancholy et al., is considered the gold standard approach, involving pressure application sufficient to achieve haemostasis while maintaining antegrade radial artery flow [4]. Despite clear evidence supporting this technique, practice variations persist across institutions, potentially compromising patient outcomes and future radial access viability [5].

Clinical supervision (CS) in nursing is a key management strategy for supporting continuous professional development and promoting the quality and safety of care [6]. It is typically defined as a formal process of professional support and learning that enables practitioners to develop knowledge and competence and to assume responsibility for their own practice in complex situations [7]. However, limited research has examined how CS can be used to support adherence to evidence‐based protocols in specialised settings such as interventional cardiology nursing.

In this context, the gap between recommended haemostasis procedures (whether manufacturer‐specified or drawn from the broader clinical evidence base) for radial artery management and actual clinical practice may reflect inadequate knowledge transfer, suboptimal supervision or organisational barriers to implementation [8, 9]. Exploring these factors at a small scale is an important step towards designing targeted strategies to improve patient safety and care quality.

This pilot study aimed to explore the alignment of clinical practices with recommended protocols for radial artery access management, alongside the strategies and characteristics of CS, in Portuguese hospital centres.

This manuscript reports the full findings of a study originally conducted as part of an academic thesis [10].

## 2. Methods

### 2.1. Study Design and Setting

A descriptive, cross‐sectional study with a quantitative design was conducted in accordance with the Strengthening the Reporting of Observational Studies in Epidemiology (STROBE) statement for observational research. The study was approved by the Ethics Committee of the Higher School of Nursing of Porto (Flow CE_34/2023) and adhered to the Declaration of Helsinki principles. Before accessing the online questionnaire, participants were presented with study information describing the aims, voluntary nature of participation, anonymity of responses and data handling procedures; informed consent was obtained electronically before questionnaire submission.

### 2.2. Participants and Sampling

Participants were recruited from five Portuguese hospital centres with established coronary 24/7 on‐call programmes, selected for their significant caseloads in interventional cardiology. Inclusion criteria comprised registered nurses working in cardiology services (inpatient units, intensive/intermediate care units, interventional cardiology laboratories or day hospitals) who provided care to patients undergoing transradial coronary procedures.

Nonprobabilistic purposive sampling using the snowball method was employed due to time constraints and accessibility considerations. At least two contact persons per institution facilitated questionnaire distribution within their networks over a 30‐day period. This recruitment strategy constitutes a convenience‐based approach and was considered appropriate for this pilot study, although it may have introduced selection bias.

### 2.3. Data Collection

Data were collected from the 30^th^ of January to the 28^th^ of February 2024 using a purpose‐designed online questionnaire administered through Microsoft Forms (Supporting Information [available here]). The instrument was developed following an extensive literature review and underwent expert content review by a panel of seven reviewers, recruited by personal acquaintance, including doctoral‐prepared nurses, clinical specialists and interventional cardiology practitioners. Because the expert panel was identified through professional networks, this convenience‐based selection is acknowledged as a methodological limitation.

The questionnaire comprised 43 items organised into three main sections:

1. Sociodemographic characteristics (10 items): age, gender, professional experience, educational qualifications and specialization.
2. Haemostasis practices (up to 24 items): protocol adherence, device usage, patient education practices and postprocedure care.
3. CS (up to 9 items): formal training, supervisory experiences, strategies employed and perceived barriers.

The haemostasis section focused particularly on the TR Band device (Terumo Interventional Systems), identified through preliminary research as the predominant method in Portuguese practice. Protocol adherence was assessed against manufacturer recommendations [11] across six key procedural components, as follows:

1. Time for first deflation (heparin ≤ 50 units/kg).
2. Time for second and subsequent deflations (heparin ≤ 50 units/kg).
3. Time for the first deflation (heparin > 50 units/kg).
4. Time for second and subsequent deflations (heparin > 50 units/kg).
5. In the event of bleeding following deflation, how to proceed.
6. After removal of the device, what care is provided to the puncture site.

This benchmark reflects the manufacturer’s instructions for use rather than an independently developed, evidence‐graded clinical guideline; where relevant, alignment with the broader patent haemostasis literature is discussed separately.

### 2.4. Data Analysis

Data were analysed using IBM SPSS version 29.0.0.0. Descriptive statistics were employed to characterise participant demographics, practice patterns and supervision experiences. Categorical variables were reported as frequencies and percentages, while continuous variables were presented as means with standard deviations or medians with ranges, as appropriate. Data normality was confirmed using Kolmogorov–Smirnov and Shapiro–Wilk tests. Given the pilot nature of the study and the sample size, exploratory subgroup comparisons were interpreted descriptively.

For exploratory descriptive subgroup analyses, we compared subgroups of nurses based on experience in cardiology (≤ 5 years versus > 5 years) and educational background (with versus without postgraduate/specialist qualifications), reflecting established competency development frameworks [12].

We used the STROBE reporting guideline [13] to draft this manuscript, and the STROBE reporting checklist [14] when editing.

## 3. Results

### 3.1. Participant Characteristics

Ninety‐six nurses participated in the study across the five hospital centres (with between 15 and 24 respondents per centre). Participants were predominantly female (65.6%, *n* = 63) with a mean age of 43.2 years (SD = 8.9, range: 26–65). Professional experience averaged 20.7 years (SD = 8.7), and median cardiology experience was 15.5 years (range: 1–34).

Educational attainment revealed that 72.9% (*n* = 70) held nursing degrees, with 27.1% (*n* = 26) having completed master’s programmes. Nearly half (49.0%, *n* = 47) were clinical specialists, while 33.3% (*n* = 32) had not pursued postgraduation studies. Medical‐surgical nursing represented the most common specialisation (30.2%), followed by rehabilitation nursing (12.5%).

Work settings were distributed among interventional cardiology laboratories (35.4%, *n* = 34), intensive/intermediate care units (39.6%, *n* = 38), general cardiology wards (24.0%, *n* = 23) and day hospitals (1.0%, *n* = 1).

### 3.2. Haemostasis Practices

#### 3.2.1. Protocol Availability and Device Usage

The majority of participants (88.5%, *n* = 85) reported having institutional haemostasis protocols, though 60% were unaware of the last revision date. All respondents using compression devices employed the TR Band system (100%, *n* = 95), with only one participant reporting manual compression use. These results suggest high protocol availability and near‐universal use of a standardised compression device, but limited awareness of protocol updating.

#### 3.2.2. Understanding of Patent Haemostasis

Only 54.7% (*n* = 52) correctly identified the concept of patent haemostasis from four multiple‐choice options. Among nurses with cardiology experience, for those with > 5 years (*n* = 75, 78.9%), understanding improved to 61.3% (*n* = 45), compared to nurses with ≤ 5 years (*n* = 20, 21.1%), whose understanding was 35% (*n* = 7). These data point to a substantial knowledge gap overall, despite higher understanding among more experienced nurses.

#### 3.2.3. Protocol Adherence

Despite 43.2% (*n* = 41) claiming to follow manufacturer recommendations, actual protocol adherence was markedly low. Only 6.3% (*n* = 6) achieved complete adherence across all six protocol components, while 44.2% (*n* = 42) demonstrated partial adherence (≥ 50% compliance). No differences in adherence appear to exist according to experience level, as nurses with > 5 years of cardiology experience achieved 42.7% (*n* = 32) rates of partial protocol compliance against less experienced colleagues (50%, *n* = 10).

The poorest performance occurred in posthaemostasis procedures, with 80.2% incorrectly applying compression bandages instead of occlusive dressings. Among nurses who correctly applied occlusive dressings, the proportion was similar between those with more than 5 years of cardiology experience (33.3%, *n* = 25) and those with 5 years or less (40%, *n* = 8), suggesting no evident difference in this practice according to experience level. Posthaemostasis care therefore emerged as a critical weakness in protocol implementation.

#### 3.2.4. Postprocedure Care Practices

Patient education practices varied considerably. Driving restrictions were recommended by 92.7% of participants, with timing preferences split between 24 h (44.8%) and 48 h (47.9%). Information delivery was predominantly verbal (67.7%), with only 24.0% providing written materials and no digital resources utilised. Overall, education was largely verbal and short‐term in focus, with minimal use of written or digital supports.

For puncture site care, 63.5% recommended compression bandages over occlusive dressings (36.5%), potentially compromising radial artery patency (Figure 1). Postprocedure follow‐up contact was rare, with only 3.1% conducting systematic patient evaluations. This limited follow‐up further restricts opportunities to reinforce best practices and detect complications.

**FIGURE 1:**
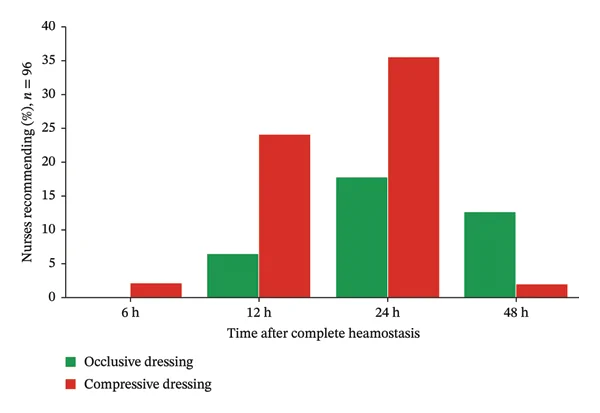
Recommended wound care after complete haemostasis by time interval (number of nurses, *n* = 96).

Exploratory subgroup analysis by educational background showed no marked descriptive differences in the main haemostasis and supervision variables between nurses with and without postgraduate/specialist qualifications; however, these comparisons should be interpreted cautiously given the pilot sample and absence of formal hypothesis testing.

### 3.3. CS Practices

#### 3.3.1. Supervisor Characteristics and Training

Most participants (88.5%, *n* = 85) lacked formal CS training, with only 10.4% having postgraduate qualifications and 1.0% holding master’s degrees in supervision. Despite limited training, 57.3% (*n* = 55) had supervised colleagues, with only 14.5% of supervisors having formal CS education. Thus, supervisory responsibilities were frequently assumed without structured preparation in CS.

#### 3.3.2. Supervisory Strategies

Supervisors predominantly employed didactic approaches: explanation (87.3%, *n* = 48) and demonstration (87.3%, *n* = 48). Interactive strategies were underutilised, with feedback employed by only 45.1% (*n* = 23) and discussion/debate by 43.1% (*n* = 22) of supervisors. From the supervisee perspective, similar patterns emerged, with demonstration (91.7%) and explanation (80.2%) most commonly experienced (Figure 2). Together, these findings suggest a supervision model that is heavily task‐oriented and less focused on reflective or dialogical learning.

**FIGURE 2:**
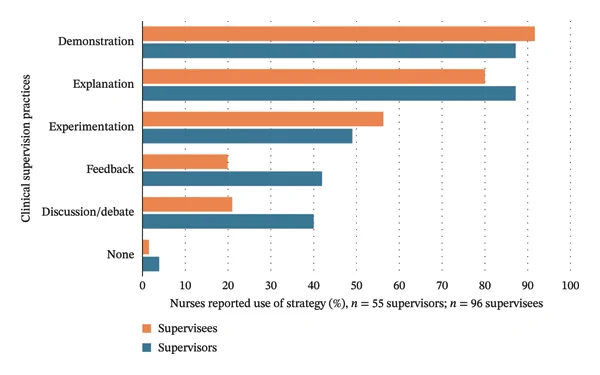
Clinical supervision practices.

#### 3.3.3. Barriers to Effective Supervision

Time constraints were identified as the primary barrier by both supervisors (56.4%) and supervisees (46.9%). High job rotation demands were the second most significant barrier (supervisors: 41.8%; supervisees: 50.0%). Limited learning opportunities and supervisor availability were also noted, though less frequently.

Competency evaluation was predominantly based on acquired competencies (76.4% of supervisors) rather than time spent or procedural numbers. This emphasis on observed competencies may be positive, but it occurs within a context of constrained supervisory time and high workload.

## 4. Discussion

This study reveals gaps between evidence‐based recommendations and actual nursing practices in radial artery haemostasis management, highlighting critical areas for improvement in CS and professional development, with repercussions for care standardisation and patient safety.

The finding that only 6.3% of participants achieved complete adherence to the TR Band manufacturer’s protocol, despite 43.2% claiming to follow manufacturer guidelines, underscores a substantial knowledge‐practice gap specific to device‐level instructions. This disparity may be related to inadequate initial training, insufficient ongoing education or institutional practice variations that deviate from evidence‐based standards [15].

The similar performance among experienced nurses (> 5 years vs. ≤ 5 years) differs from Benner’s competency development model, where it is suggested that expertise develops through sustained exposure and reflection [12]. However, the persistent knowledge gaps even among experienced practitioners indicate that experience alone is insufficient to ensure evidence‐based practice without structured guidance and supervision.

The limited understanding of patent haemostasis (54.2%) is particularly concerning, given its fundamental importance in preventing radial artery occlusion [4]. Patent haemostasis prevents radial artery occlusion while achieving adequate haemostasis, preserving future access options for patients who may require repeat procedures [5]. The knowledge deficit in this area suggests inadequate dissemination of evidence‐based practices and highlights the need for targeted educational interventions.

The substantial variation in postprocedure care recommendations, particularly regarding puncture site management, reflects the absence of standardised, evidence‐based protocols. The preference for compression bandages over occlusive dressings (63.5% vs. 36.5%) may inadvertently compromise radial artery patency, as compression can occlude arterial flow even after successful haemostasis [16].

The limited use of written (24.0%) or digital educational materials represents a missed opportunity for patient engagement and safety. Contemporary healthcare emphasises patient‐centred care and shared decision‐making, necessitating comprehensive, accessible patient education resources [17]. The rarity of postprocedure follow‐up (3.1%) further compromises patient safety monitoring and continuous care quality improvement. This points to the need to implement nursing discharge planning programs.

The predominance of didactic supervision strategies (explanation and demonstration: 87.3%) over interactive approaches (feedback: 45.1%; discussion: 43.1%) suggests a traditional, hierarchical supervision model that may limit critical thinking development and evidence‐based practice adoption [18]. Effective CS should balance formative, normative and restorative functions, employing diverse strategies tailored to individual learning needs and practice contexts [19].

The lack of formal CS training among supervisors (88.5%) represents a significant system‐level deficit. Without appropriate pedagogical preparation, supervisors may inadvertently perpetuate suboptimal practices or fail to identify knowledge gaps requiring correction [20]. The SafeCare supervision model, developed specifically for Portuguese nursing contexts, offers a structured framework that could address these deficits through systematic implementation [3].

The identification of time constraints and high job rotation as primary supervision barriers reflects broader healthcare system challenges. These findings align with international literature identifying organisational factors as significant impediments to effective CS [21]. Without institutional commitment to protected supervision time and stable staffing patterns, supervision quality will remain compromised.

Several limitations should be acknowledged. The nonprobabilistic sampling method limits generalisability to the broader Portuguese nursing population. The cross‐sectional design prevents causal inference regarding supervision‐practice relationships. Self‐reported data may be subject to social desirability bias, potentially overestimating positive practices. The focus on Portuguese centres may limit international applicability, though similar healthcare system challenges suggest broader relevance. In addition, the expert validation panel was recruited through professional acquaintance, which may have limited the diversity of perspectives included in the instrument review.

### 4.1. Implications for Nursing Management

These findings point to several practical considerations for nursing management, without implying that any single intervention has been shown to improve outcomes in this study. Supervisor preparation could be strengthened through structured, competency‐based training programmes, given that most supervisors in this sample lacked formal CS education. Such training programmes exist in Portugal as postgraduations, as well as Master’s degrees, for example. Protected time for supervision may need to be explicitly built into staffing schedules, since time constraints were identified as the most frequently reported barrier by both supervisors and supervisees. Competency assessment approaches could be reviewed to ensure they consistently capture observed practice rather than relying solely on time‐based or procedural‐count criteria. Protocol governance structures may benefit from regular review and clearer institutional dissemination, given the low rates of full adherence to the device manufacturer’s protocol observed here. Routine audit and feedback mechanisms could help identify and address practice variation in haemostasis management on an ongoing basis. Finally, staffing stability, particularly reducing high job rotation, may support continuity in both supervisory relationships and practice consistency, though this study’s cross‐sectional design does not permit conclusions about the effectiveness of any of these measures.

## 5. Conclusion

This study demonstrates substantial variability in adherence to evidence‐based protocols for radial artery haemostasis management among Portuguese nurses, highlighting significant opportunities for practice improvement. While institutional protocols were widely available and the TR Band system was used almost universally, awareness of protocol updates and understanding of the patent haemostasis concept were limited, and complete protocol adherence was achieved by only a small minority of participants. Posthaemostasis care, particularly the choice between compression bandages and occlusive dressings, emerged as the area of poorest performance and represented a critical weakness in protocol implementation. Contrary to expectations, adherence and posthaemostasis practice did not differ meaningfully according to years of cardiology experience, although understanding of patent haemostasis was somewhat higher among more experienced nurses.

The predominance of didactic supervision approaches over interactive, reflective strategies was identified alongside the observed gaps in haemostasis protocol adherence. While the broader literature discusses CS as a potential facilitator of evidence‐based practice, the cross‐sectional design of this study does not allow inference that supervision practices directly influenced haemostasis protocol adherence in this sample.

The main contribution of this pilot study is to describe, in parallel, the current state of haemostasis protocol adherence and CS practices among nurses caring for patients undergoing transradial procedures. Healthcare organisations may wish to consider the development of clear supervision frameworks and reinforcement of evidence‐based protocols as separate, complementary areas warranting further research in interventional cardiology settings.

## Funding

This article was supported by National Funds through FCT–Fundação para a Ciência e a Tecnologia,I.P., within RISE‐Health, R&D Unit (reference UID/06397/2025).

## Disclosure

The authors wish to disclose that this manuscript represents the full report of a study whose preliminary findings were derived from an academic thesis and previously presented as a conference abstract at the ACNAP 2025 congress and published in the *European Journal of Cardiovascular Nursing* [22]. The present manuscript expands on this prior thesis‐derived and conference‐disseminated work by providing a complete analysis and discussion of the study’s methods, results and clinical implications.

## Conflicts of Interest

The authors declare no conflicts of interest.

## Supporting Information

Additional supporting information can be found online in the Supporting Information section.

## Supporting information


**Supporting Information 1** “Nursing Practices in Radial Artery Haemostasis after Coronary Angiography” online questionnaire file.

**Supporting Information 2** STROBE_checklist_filled_final.

## Data Availability

The data that support the findings of this study are available from the corresponding author upon reasonable request.
