# Supplementary material for: Clinical Supervision in Nursing and Evidence‐Based Practices in Radial Haemostasis: A Pilot Study
Source: J Nurs Manag. 2026 Oct 15;2026:6108309. doi: 10.1155/jonm/6108309 (PMC13670135; doi:10.1155/jonm/6108309)
Supplement: Supplementary file 1 [file JONM-2026-6108309-s001.docx]

STROBE Statement—Checklist of items that should be included in reports of ***cross-sectional studies***

|  | Item No | Recommendation |
| --- | --- | --- |
| **Title and abstract** | 1 | Page 1, Title: identified as a 'pilot study'; Abstract states 'A descriptive, cross-sectional study'. |
|  |  | Abstract (Methods and Results, Conclusion) summarises design, sample (n=96 nurses), key haemostasis and supervision findings, and main conclusions. |
| Introduction | | |
| Background/rationale | 2 | Introduction, para 5 (last paragraph): study aims explicitly stated as exploring alignment of clinical practice with protocols and role of clinical supervision. |
| Objectives | 3 | Introduction, final paragraph: study aims stated; no prespecified hypotheses were formulated given the pilot/exploratory nature. |
| Methods | | |
| Study design | 4 | Methods, 'Study Design and Setting': descriptive, cross-sectional, quantitative design stated early, following STROBE guidance. |
| Setting | 5 | Methods, 'Study Design and Setting' and 'Data Collection': ethics approval, five Portuguese hospital centres, and data collection dates (28 January to 28 February 2024) reported. |
| Participants | 6 | Methods, 'Participants and Sampling': inclusion criteria (registered nurses in cardiology services caring for transradial patients) and non-probabilistic purposive/snowball sampling method described. |
| Variables | 7 | Methods, 'Data Collection': outcomes (haemostasis practices, protocol adherence, supervision strategies) and predictors (experience, education) defined via the 43-item questionnaire; no formal diagnostic criteria applicable. |
| Data sources/ measurement | 8* | Methods, 'Data Collection': single self-report online questionnaire (Microsoft® Forms) used for all participants; no comparison of multiple assessment methods, as only one instrument was administered. |
| Bias | 9 | Methods, 'Participants and Sampling' and Discussion 'Limitations': convenience/snowball sampling and expert-panel recruitment via personal acquaintance acknowledged as potential sources of selection bias. |
| Study size | 10 | Not explicitly reported; sample size (n=96) reflects convenience/purposive recruitment over a fixed 30-day/1-month collection period rather than a pre-calculated target (pilot study). |
| Quantitative variables | 11 | Methods, 'Data Analysis': continuous variables (age, experience) presented as means/SD or medians/ranges; categorical variables (protocol adherence, ≤5 vs >5 years experience) reported as frequencies/percentages, as stated. |
| Statistical methods | 12 | (a) Methods, 'Data Analysis': descriptive statistics (frequencies, percentages, means/SD, medians/ranges) described; no confounder adjustment performed, consistent with the descriptive design. |
|  |  | (b) Methods, 'Data Analysis': exploratory descriptive subgroup comparisons by cardiology experience (≤5 vs >5 years) and educational background described. |
|  |  | Not reported; the questionnaire required responses to all items, and no missing-data handling procedure is described. |
|  |  | Not applicable; convenience/purposive snowball sampling was used rather than a probability sampling strategy requiring analytical weighting. |
|  |  | Not performed; no sensitivity analyses are reported, consistent with the descriptive, exploratory nature of this pilot study. |
| Results | | |
| Participants | 13* | (a) Results, 'Participant Characteristics': 96 nurses across five hospital centres (15-24 respondents per centre) included and analysed; no separate eligibility/non-completion stages reported. |
|  |  | Not reported; reasons for non-participation among nurses invited via snowball distribution were not captured, a limitation of the online/anonymous recruitment method. |
|  |  | Not applicable/not included; given the single-stage online questionnaire design, a participant flow diagram was not used. |
| Descriptive data | 14* | (a) Results, 'Participant Characteristics': demographic and professional characteristics reported (sex, age, experience, education, specialisation, work setting). |
|  |  | Not explicitly reported; the online questionnaire required responses to all items, so item-level missing data were not separately quantified. |
| Outcome data | 15* | Results, 'Haemostasis Practices' and 'Clinical Supervision Practices' sections: outcome frequencies reported (e.g., 6.3% full protocol adherence; 54.2% correct patent haemostasis understanding; 88.5% lacking formal CS training). |
| Main results | 16 | (a) Results present unadjusted frequencies/percentages only (e.g., 76.2% partial compliance among experienced nurses); no confounder-adjusted estimates or confidence intervals are reported, consistent with the descriptive design. |
|  |  | Not applicable; continuous variables (e.g., age, experience) were not categorised into ordinal groups for the main results beyond the ≤5/>5-year experience split used for subgroup description. |
|  |  | Not applicable; the study reports absolute frequencies/percentages rather than relative risk estimates. |
| Other analyses | 17 | Results, para after Figure 1 (subgroup analysis paragraph): exploratory descriptive subgroup analysis by educational background reported, noting no marked differences versus postgraduate/specialist qualifications, interpreted cautiously. |
| Discussion | | |
| Key results | 18 | Discussion, first paragraph: key results summarised in relation to the study objectives (knowledge-practice gap in radial haemostasis and clinical supervision role). |
| Limitations | 19 | Discussion, 'Several limitations should be acknowledged' paragraph: non-probabilistic sampling, cross-sectional design, self-report/social desirability bias, single-country focus, and convenience-based expert panel recruitment discussed as limitations. |
| Interpretation | 20 | Discussion paragraphs throughout (e.g., on experience, patent haemostasis, supervision strategies) offer cautious, hedged interpretation ('may be related to', 'suggests', 'may help support'), considering the pilot design and prior literature. |
| Generalisability | 21 | Discussion, limitations paragraph: 'The focus on Portuguese centres may limit international applicability, though similar healthcare system challenges suggest broader relevance.' |
| Other information | | |
| Funding | 22 | FUNDING section: "This research was conducted as part of a Master's degree programme at Higher Nursing School of Porto and received no external funding." ACKNOWLEDGMENTS section additionally discloses that preliminary findings from this same study and dataset were previously presented as a conference abstract at ACNAP 2025 and published in the European Journal of Cardiovascular Nursing (ref. 20). |

*Give information separately for exposed and unexposed groups.

**Note:** An Explanation and Elaboration article discusses each checklist item and gives methodological background and published examples of transparent reporting. The STROBE checklist is best used in conjunction with this article (freely available on the Web sites of PLoS Medicine at http://www.plosmedicine.org/, Annals of Internal Medicine at http://www.annals.org/, and Epidemiology at http://www.epidem.com/). Information on the STROBE Initiative is available at www.strobe-statement.org.
