# Supplementary material for: Clinical Supervision in Nursing and Evidence‐Based Practices in Radial Haemostasis: A Pilot Study
Source: J Nurs Manag. 2026 Oct 15;2026:6108309. doi: 10.1155/jonm/6108309 (PMC13670135; doi:10.1155/jonm/6108309)
Supplement: Supplementary file 2 [file JONM-2026-6108309-s002.docx]

Nursing Practices in Radial Artery Haemostasis after Coronary Angiography

Dear Nurse,

I am writing to invite you to take part in the study: "Nursing Practices in Radial Artery Haemostasis after Coronary Angiography: A Clinical Supervision Problem?" It is important that you are aware of the aims of the study, any risks or inconveniences of participation, the conditions under which it is conducted, and the implications for you.

You are entirely free to take part or not. You may therefore decline to participate without this affecting you in any way.

The purpose of this document is to provide you with all the information you may need, including how your personal data will be used or disclosed to third parties should you choose to participate. Please read this document carefully.

If you do not understand any of the information in this document, or if you have any doubts regarding the nature of the study or your participation in it, please ask the study coordinator (contact details at the end of the document) to provide further information or clarify any queries you may have.

**STUDY: Nursing Practices in Radial Artery Haemostasis after Coronary Angiography: A Clinical Supervision Problem?**

## SCOPE OF THE STUDY

This study forms part of the curriculum of the Master's Degree in Clinical Supervision at the Higher School of Nursing of Porto, supervised by Professor Paulo Marques, PhD, co-supervised by Professor Cristina Barroso, PhD, and conducted under the responsibility of the Master's student David Rodrigues.

## PURPOSE AND DESCRIPTION OF THE STUDY

This study aims to identify nursing practices relating to radial artery haemostasis following coronary angiography, as well as to determine the knowledge base and clinical supervision strategies used for the initial and ongoing training of nurses who provide care to patients undergoing coronary angiography via the radial artery. Participants will be invited to take part via an electronic questionnaire. If you decide to take part, you will be asked to complete a questionnaire that takes approximately 5-10 minutes.

Information on the use and protection of personal data, and availability for clarifying any queries, can be found at http://tinyurl.com/4pxa4ez9

*Yes / No*

**1. Having read the above information, do you agree to take part in the study "Nursing Practices in Radial Artery Haemostasis following Coronary Angiography: A Clinical Supervision Issue?" and, in doing so, do you authorise the processing of the personal data collected here? ***

- Yes
- No (End of questionnaire: “Thank you for your participation!”)

# Sociodemographic Characteristics

**2. Sex ***

- Female
- Male

**3. Age (years) ***

*The number must be between 20 and 70*

**4. Years of experience as a nurse ***

*The number must be between 1 and 40*

**5. Years of experience in cardiology/Cardiac Intensive Care Unit (CICU)/interventional cardiology/cardiology day hospital ***

*The number must be between 1 and 40*

**6. Main work area (where you work the majority of your hours) ***

- Inpatient ward
- Cardiology intermediate/intensive care unit
- Interventional cardiology
- Day hospital

**7. Main institution where you work ***

- São João University Hospital Centre
- Santo António University Hospital Centre
- Vila Nova de Gaia/Espinho Hospital Centre
- Tâmega e Sousa Hospital Centre
- Coimbra University Hospital, CHUC-HUC

**8. Academic qualifications ***

- Nursing degree
- Master's degree
- Doctoral degree

**9. Other qualifications ***

- Postgraduate qualification
- Specialisation
- None
- Other

# Specialisation

**10. Please indicate the specialisation(s) you hold: ***

- None
- Medical-Surgical Nursing
- Medical-Surgical Nursing, in the area of Nursing Care for the Person in Palliative Care
- Medical-Surgical Nursing, in the area of Nursing Care for the Person with Chronic Illness
- Medical-Surgical Nursing, in the area of Nursing Care for the Person in Critical Condition
- Medical-Surgical Nursing, in the area of Nursing Care for the Person in the Perioperative Setting
- Rehabilitation Nursing
- Child Health and Paediatric Nursing
- Maternal and Obstetric Health Nursing
- Mental Health and Psychiatric Nursing
- Community Nursing
- Community Nursing, in the area of Community Health and Public Health Nursing
- Community Nursing, in the area of Family Health Nursing

# Type of Care Provided

**11. Do you provide care to patients undergoing interventional cardiology/haemodynamic procedures via the radial artery? ***

- Yes
- No (End of questionnaire: “Thank you for your participation!”)

# Haemostasis Protocol

**12. Does your centre have a haemostasis protocol for radial access following cardiac catheterisation? ***

- Yes
- No (Go to: Haemostasis Method”)

**13. When was it last reviewed? (if "Other", please indicate the approximate year)**

- Never
- Don't know
- Other

# Haemostasis Method

**14. At your centre, what is the most commonly used haemostasis method for radial access? ***

- Manual compression (Go to: “Manual Compression”)
- Compression device (Go to: “Radial Compression device”)
- Compressive dressing (Go to: “Compressive dressing”)

# Manual Compression

*Select the option that most closely resembles your practice*

**15. Once the introducer sheath is removed, the puncture site occluded with folded gauze, and pressure applied: ***

- Minimum NECESSARY to prevent bleeding for five minutes; apply a compressive dressing.
- Minimum NECESSARY to prevent bleeding for 10 (ten) minutes; apply a compressive dressing.
- Minimum NECESSARY to prevent bleeding for 15 (fifteen) minutes; apply a compressive dressing.
- Minimum necessary to prevent bleeding for five minutes; apply an OCCLUSIVE dressing.
- Minimum necessary to prevent bleeding for 10 (ten) minutes; apply an OCCLUSIVE dressing.
- Minimum necessary to prevent bleeding for 15 (fifteen) minutes; apply an OCCLUSIVE dressing.
- Maximum TOLERATED by the patient for five minutes; apply a compressive dressing.
- Maximum TOLERATED by the patient for 10 (ten) minutes; apply a compressive dressing.
- Maximum TOLERATED by the patient for 15 (fifteen) minutes; apply a compressive dressing.
- Maximum tolerated by the patient for five minutes; apply an OCCLUSIVE dressing.
- Maximum tolerated by the patient for 10 (ten) minutes; apply an OCCLUSIVE dressing.
- Maximum tolerated by the patient for 15 (fifteen) minutes; apply an OCCLUSIVE dressing.

# Radial Compression Device

**16. Please indicate the device most commonly used at your centre ***

- TR-BAND (Terumo)
- VASOBAND (VASOInnovations)
- Safeguard Radial (Merit Medical)
- Radistop (Abbott)
- TRAcelet (Medtronic)
- RadAR™ Vascular Compression Device (Advanced Vascular Dynamics)
- Zephyr® Vascular Compression Device (Advanced Vascular Dynamics)
- Bengal radial compression band (Ates Group)
- Radial Artery Compression Tourniquet (Demax Medical)
- VasoStat haemostasis device (Forge Medical Inc)
- PreludeSYNC EZ™ Radial Compression Device (Merit Medical Systems Inc)
- PreludeSYNC EVO™ Radial Compression Device (Merit Medical Systems Inc)
- D-Stat® Rad-Band Topical Haemostat (Teleflex)
- ROC band (TZ Medical)

# Patent Haemostasis

The concept of patent haemostasis has been described since 2012.

**17. Which of the following options best describes the concept of "patent haemostasis"? ***

- Inflate to the maximum pressure permitted by the device, unless peripheral cyanosis/pain occurs.
- Apply sufficient pressure to prevent bleeding, while confirming the presence of an ulnar pulse.
- Inflate to the device's nominal pressure, with the first deflation performed as early as possible.
- Inflate only to the minimum pressure required to prevent bleeding, while maintaining radial flow. (Correct answer)

**18. At your centre, how is the inflation procedure for the TR-Band defined? ***

- Standard approach (the same for all patients).
- According to manufacturer instructions.
- According to criteria established by the institution.

# TR-Band Inflation

**19. What is the main criterion used for determining the volume of air inflated into the TR-Band upon placement? ***

- Dose of heparin used during the procedure
- Patient's blood pressure
- Circumference of the patient's distal forearm
- Barbeau test or pulse oximetry waveform analysis
- Allen/modified Allen test
- Local pain
- Bleeding
- Other

# Haemostasis Protocol

**20. For a diagnostic catheterisation, what is the stipulated time for the first deflation? ***

- 30 minutes
- 60 minutes (1h)
- 90 minutes (1h30)
- 120 minutes (2h)
- 150 minutes (2h30)

**21. Following the first deflation, when does the second deflation (and subsequent ones) typically occur? ***

- 10 minutes
- 15 minutes
- 30 minutes
- 45 minutes
- 60 minutes

**22. For a therapeutic catheterisation/percutaneous coronary intervention (PCI), what is the stipulated time for the first deflation? ***

- 60 minutes (1h)
- 90 minutes (1h30)
- 120 minutes (2h)
- 150 minutes (2h30)
- 180 minutes (3h)

**23. Following the first deflation, when does the second deflation (and subsequent ones) typically occur? ***

- 15 minutes
- 30 minutes
- 45 minutes
- 60 minutes
- 90 minutes

**24. In the event of bleeding following deflation, how do you proceed? ***

- Re-inflate with the same volume of air previously withdrawn and restart the protocol.
- Re-inflate with the same volume of air previously withdrawn and continue the protocol.
- Re-inflate with sufficient air to prevent bleeding and restart the protocol.
- Re-inflate with sufficient air to prevent bleeding and continue the protocol.
- Remove the haemostatic band and perform manual compression.

# Puncture Site

**25. After removal of the compression device, what care do you provide to the puncture site? ***

- Cleansing with warm saline and application of an occlusive dressing.
- Cleansing with warm saline and application of a compressive dressing.
- Cleansing with antiseptic and application of an occlusive dressing.
- Cleansing with antiseptic and application of a compressive dressing.

# Compressive Dressing

**26. How is the compressive dressing performed? ***

- Adhesive tape (e.g., Mefix®) over folded gauze, around the wrist.
- Adhesive tape (e.g., Mefix®) over folded gauze, in a cross pattern over the puncture site.
- Adhesive elastic bandage (e.g., Tensoplast) over folded gauze, around the wrist.
- Adhesive elastic bandage (e.g., Tensoplast) over folded gauze, in a cross pattern over the puncture site.
- Adhesive tape (e.g., Mefix®) over folded gauze, around the wrist; radial flow verified using the reverse Barbeau test/plethysmography.
- Adhesive tape (e.g., Mefix®) over folded gauze, in a cross pattern over the puncture site; radial flow verified using the reverse Barbeau test/plethysmography.
- Adhesive elastic bandage (e.g., Tensoplast®) over folded gauze, around the wrist; radial flow verified using the reverse Barbeau test/plethysmography.
- Adhesive elastic bandage (e.g., Tensoplast®) over folded gauze, in a cross pattern over the puncture site; radial flow verified using the reverse Barbeau test/plethysmography.

# Discharge Preparation

**27. Are recommendations given to patients following the procedure regarding care and monitoring of the access site?**

- Yes
- No

What recommendations are given to patients following the procedure regarding care and monitoring of the access site?

**28. Driving: ***

- Driving restriction for 24h
- Driving restriction for 48h
- Other

**29. In the event of pain, paraesthesia/dysaesthesia, or signs of inflammation: ***

- Call SNS24 (National TeleHealth Line)
- Contact the hospital
- Attend a Health Centre
- Attend the Emergency Department
- Call 112
- Other

**30. In the event of bleeding: ***

- Call SNS24 (National TeleHealth Line)
- Contact the hospital
- Attend a Health Centre
- Attend the Emergency Department
- Call 112
- Other

**31. Exertion/load on the treated limb: ***

- No weight-bearing on the treated limb for 6h
- No weight-bearing on the treated limb for 12h
- No weight-bearing on the treated limb for 24h
- No weight-bearing on the treated limb for 48h
- Other

**32. Care of the puncture site ***

- Keep occlusive dressing on for 12h
- Keep occlusive dressing on for 24h
- Keep occlusive dressing on for 48h
- Keep compressive dressing on for 4h
- Keep compressive dressing on for 6h
- Keep compressive dressing on for 12h
- Keep compressive dressing on for 24h
- Keep compressive dressing on for 48h
- Other

**33. How is this information provided? ***

- In writing (leaflet, brochure...)
- Verbally
- Digital format
- Other

**34. Who receives this information? ***

- Patient
- Family member/caregiver

**35. Is a post-procedure assessment carried out after discharge (e.g., remote nursing consultation)? ***

- Yes, up to 48h post-procedure
- Yes, up to 7 days post-procedure
- No

# Clinical Supervision in Nursing

**36. Please indicate the type of training you have in clinical supervision in nursing: ***

- Postgraduate qualification
- Master's degree
- Doctoral degree
- None

**37. Is there an institutional protocol for the induction of new nurses that includes radial artery haemostasis practices following coronary angiography? ***

- Yes
- No

**38. Have you ever taken part in the induction of a colleague that included radial artery haemostasis practices following coronary angiography? ***

- Yes
- No

# Supervisory Practices - Supervisor

**39. As a supervisor, please indicate whether you use any of the following supervisory practices: ***

- Demonstration
- Explanation
- Hands-on practice
- Feedback
- Discussion/debate
- None of the above

**40. The supervisee's competency is assessed mainly on the basis of: ***

- Length of induction period
- Number of procedures
- Competencies acquired

**41. During the induction period, please indicate the factor(s) that typically limit autonomous and safe practice in radial artery haemostasis: ***

- Short induction period
- Limited learning opportunities
- Limited availability as a supervisor
- High job rotation
- Other

# Supervisory Practices - Supervisee

**42. During your induction, which supervisory practices were used? ***

- Demonstration
- Explanation
- Hands-on practice
- Feedback
- Discussion/debate
- None of the above

**43. Your competency was assessed mainly on the basis of: ***

- Length of induction period
- Number of procedures
- Competencies acquired

**44. During the induction period, please indicate the factor(s) that typically limit autonomous and safe practice in radial artery haemostasis: ***

- Short induction period
- Limited learning opportunities
- Limited availability of the supervisor
- High job rotation
- Other

# Thank you for your participation!

**45. Would you like to add any further information/comments?**
